# Expression and Purification of Human Membrane Progestin Receptor α (mPRα)

**DOI:** 10.1371/journal.pone.0138739

**Published:** 2015-09-23

**Authors:** Md. Babul Hossain, Takayuki Oshima, Shizuka Hirose, Jun Wang, Toshinobu Tokumoto

**Affiliations:** 1 Integrated Bioscience Section, Graduate School of Science and Technology, National University Corporation Shizuoka University, Ohya 836, Suruga-ku, Shizuoka, 422–8529, Japan; 2 Department of Biological Science, Faculty of Science, National University Corporation Shizuoka University, Ohya 836, Suruga-ku, Shizuoka, 422–8529, Japan; USDA-ARS, UNITED STATES

## Abstract

Membrane progestin receptors (mPRs) are responsible for mediating the rapid, nongenomic activity of progestins and belong to the G protein-coupled receptor (GPCR) family. mPRs are also considered as attractive proteins to draw a new medicinal approach. In this study, we optimized a procedure for the expression and purification of recombinant human mPRα protein (hmPRα) by a methylotropic yeast, *Pichia pastoris*, expression system. The protein expressed in crude membrane fractions exhibited a binding affinity of Kd = 3.8 nM and Bmax = 288.8 fmol/mg for progesterone. These results indicated that the hmPRα expressed in yeast was active. Solubilized hmPRα was purified through three column chromatography steps. A nickel-nitrilotriacetic acid (Ni-NTA) column was first used, and the mPRα proteins were then bound to cellulose resin with free amino groups (Cellufine Amino) and finally passed through an SP-Sepharose column. The optimization of expression and purification conditions resulted in a high yield of purified hmPRα (1.3–1.5 mg from 1 L culture). The purified hmPRα protein demonstrated progesterone binding (Kd = 5.2 nM and Bmax = 111.6 fmol/mg). The results indicated that we succeeded in solubilizing and purifying hmPRα in an active form. Sufficient amount of active hmPRα protein will support the establishment of applications for the screening of ligands for mPRα.

## Introduction

Progestins act as a key regulating factor in controlling the reproductive tissues. Progesterone was identified as a natural progestin in the human body [1]. Synthetic progestins have been produced and are frequently used for medical purposes. Progesterone is a well-known steroid that is produced by the ovary depending on the physiological conditions of the ovary and gonadotropin levels [2]. Progesterone generates a number of physiological effects in different tissues through various mediating mechanisms in each tissue. Although the physiological effects of progesterone have been known to be mediated by the regulation of gene expression associated with nuclear progesterone receptors [3], new insight on the activity of progesterone was provided by the identification of membrane progestin receptors (mPRs) [4]. Certain nongenomic effects of progestins, such as oocyte maturation, are mediated by mPRs on the plasma membrane and induce rapid intracellular changes. Oocyte maturation-inducing steroids (MISs) are produced in response to luteinizing hormone (LH) in the follicular envelope in fish [5]. This progestin-induced nongenomic activity in oocytes mediated by mPR causes the cells to proceed through meiotic cell cycles [6–10]. Based on phylogenetic analysis, mPRs can be categorized into a new protein family of G protein-coupled receptors (GPCRs), the PAQR (progestin and adipoQ receptors) family [11]. This family contains three subordinate types termed mPRα, β and γ (corresponding to PAQR7, 8 and 5, respectively) [12]. Based on the analysis of proteins expressed in human breast cancer cells, PAQR6 and 9 were also categorized as new subtypes of mPR (mPRδ and ε, respectively) [13]. The expression of mPR mRNAs has been observed in reproductive tissues (ovary, testis, uterus and placenta) and in nonreproductive tissues (kidney, brain and intestinal tissues) in the human body [9]. The broad distribution of mPRs in different tissues suggests that mPRs perform various functions in a large range of target tissues. Additionally, mPRs could serve as a target for endocrine-disrupting chemicals (EDCs). Although the distinct role of mPRs remains under investigation, mPRs could be a target for a novel class of pharmaceuticals and EDCs. Thus, we aimed to produce a recombinant mPR protein.

The methylotrophic yeast *Pichia pastoris* is an efficient host for the expression of membrane proteins [14,15] and secretory proteins [16–19]. Recently, research has been conducted on human histamine H1 receptor and GPCR expression by *P*. *pastoris* [15]. Previously we reported the expression of mPR protein in human cancer cell lines and in *Escherichia coli* [20]. The large-scale culturing of *E*. *coli* is possible but did not produce an active form of recombinant mPR [20]. In addition, the mPR expression levels in mammalian cells were extremely low and did not generate a sufficient amount of protein for purification, structural and biochemical analysis. More than one hundred reports have emphasized the expression of GPCRs and their large-scale purification using *P*. *pastoris*. Natural ligand binding has been assessed by the expression of mammalian GPCRs in *P*. *pastoris* [21,22]. *P*. *pastoris* has been widely used for the expression of GPCRs. The structures of two human GPCRs (the histamine H1 and the adenosine A_2a_ receptor) were determined using recombinant protein expressed in *P*. *pastoris* [15,23]. Thus we selected *P*. *pastoris* for the expression of large amounts of mPR.

Previously, we established a procedure for producing and purifying recombinant goldfish mPRα, but this method generates very low amounts of protein [24]. In this study, we established a yeast strain for the expression of human mPRα (hmPRα) according to the method for goldfish mPRα and optimized the conditions to obtain large amounts of product. Through the optimization of culture conditions, homogenization protocol and conditions for column chromatography, we established a procedure to obtain relatively a large amount of hmPRα. This evolving new procedure could be used to produce sufficient amounts of hmPRα protein to develop a screening system for mPRα-affecting agents or to determine the structure of mPRα.

## Results

### 1. Recombinant human mPRα protein expression

**Fig 1:**
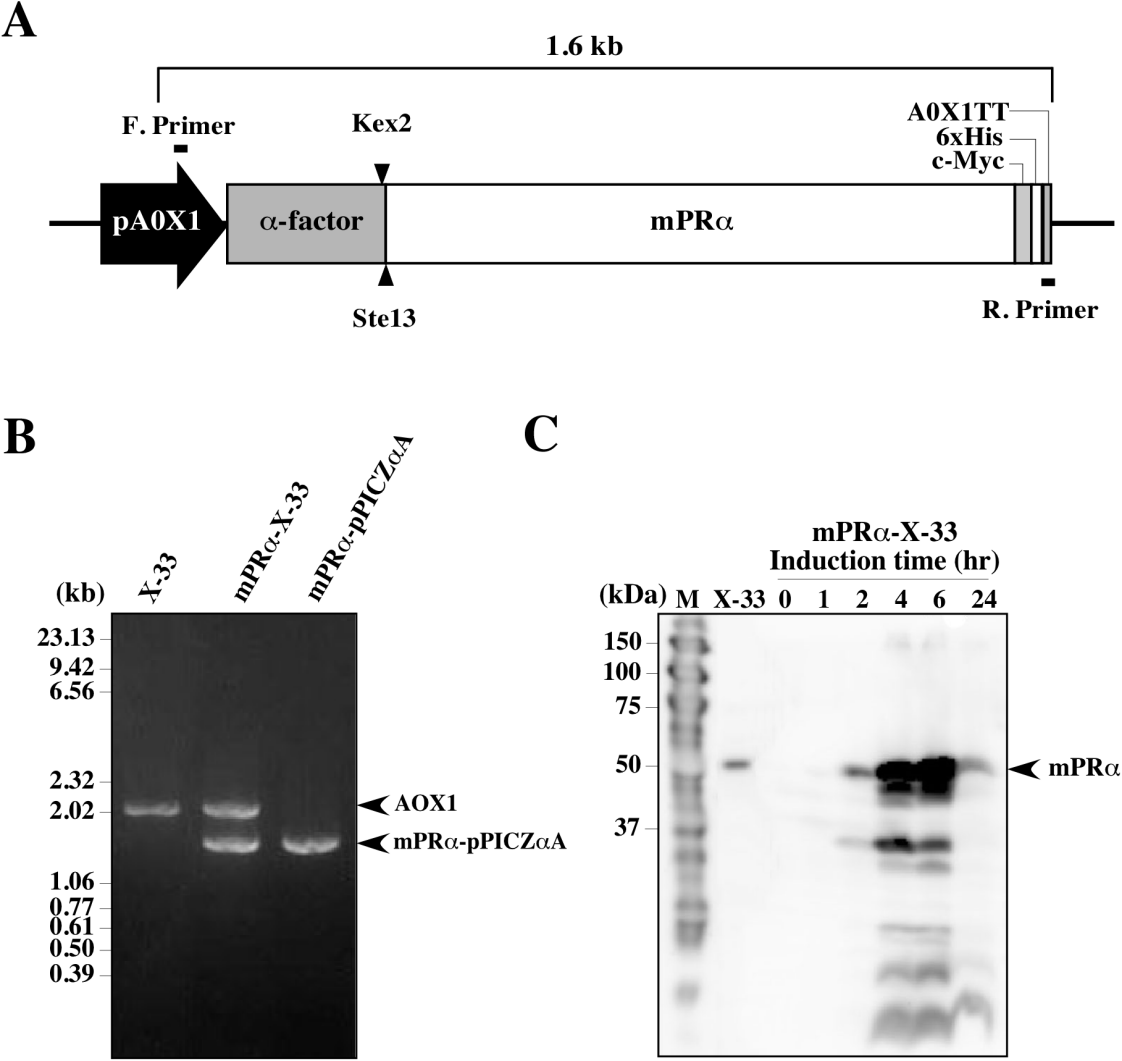
Expression of human mPRα in *Pichia pastoris*. **(A)** Schematic representation of the hmPRα expression cassette that was inserted into the yeast cells to produce mPRα protein. The fusion peptide consisted of hmPRα, a α-factor signal sequence, a C-terminal histidine (6x His), and a c-Myc epitope controlled by the methanol-inducible AOX1 promoter (pAOX1) and the AOX1 transcription termination region (AOX1 TT). The black bars above and below the cassette indicate the 5′AOX1 (F. primer) and 3′AOX1 (R. primer) primer binding sites, respectively. The AOX1 gene of the yeast cells remained within the expression cassette (2.1 kbp). **(B)** Gene insertion was verified by PCR. DNA fragments were amplified using genome DNA from untransformed yeast cells (X-33), genome DNA from hmPRα-transformed cells (mPRα-X-33) or transformed vector DNA (mPRα-pPICZαA) as templates. **(C)** After protein expression induction in culture with methanol, samples were taken at 0, 1, 2, 4, 6 and 24 hours. Expression of hmPRα was determined by western blot analysis. A protein band of 50 kDa was reacted with anti-His-tag antibody in the extract prepared from hmPRα-transformed cells (mPRα-X-33).

Wild yeast *P*. *pastoris* was used for the expression and production of recombinant hmPRα protein. For expression, the cDNA of hmPRα was fused to the secretion signal sequence of the α-factor from *S*. *cerevisiae* in the expression cassette (Fig 1A). The construct was inserted into the host yeast genome by homologous recombination. The successful insertion of the cassette, along with its promoter and terminator that control the transcription of the heterologous mPRα gene fusion, into the yeast cells was confirmed by PCR using *AOX1* primer sets (Fig 1B).

The expressed fusion hmPRα protein carried a c-Myc epitope and a histidine tag (His-tag) on its C-terminal end. Expression was induced by the presence of 0.5% methanol in the BMMY medium. The expression of hmPRα protein was confirmed by western blot analysis (Fig 1C). The protein band of approximately 50 kDa was detected. The theoretical molecular mass of hmPRα containing a α-factor signal peptide is approximately 52 kDa, which was consistent with the molecular mass of the detected band.

To determine the optimal conditions for the expression of hmPRα, 1 mL aliquots of the culture were collected after 0, 1, 2, 4, 6 and 24 hours, and the amount of expressed hmPRα was analyzed. The highest level of hmPRα expression was detected at 6 hours (Fig 1C). The optimal cell density before the initiation of induction was also examined. When the cell density increased, the yield of hmPRα protein also increased. After several trials, we found that a cell density of OD_600_ 21–23 during methanol induction was optimal, in contrast to previous conditions established for goldfish mPRα protein production at an OD_600_ of 1.0–3.0 [24]. Therefore, we succeeded in producing the hmPRα protein at higher cell densities.

### 2. Specific binding of [^3^H]1,2,6,7-progesterone on plasma membranes prepared from hmPRα-expressed *P*. *pastoris*

**Fig 2:**
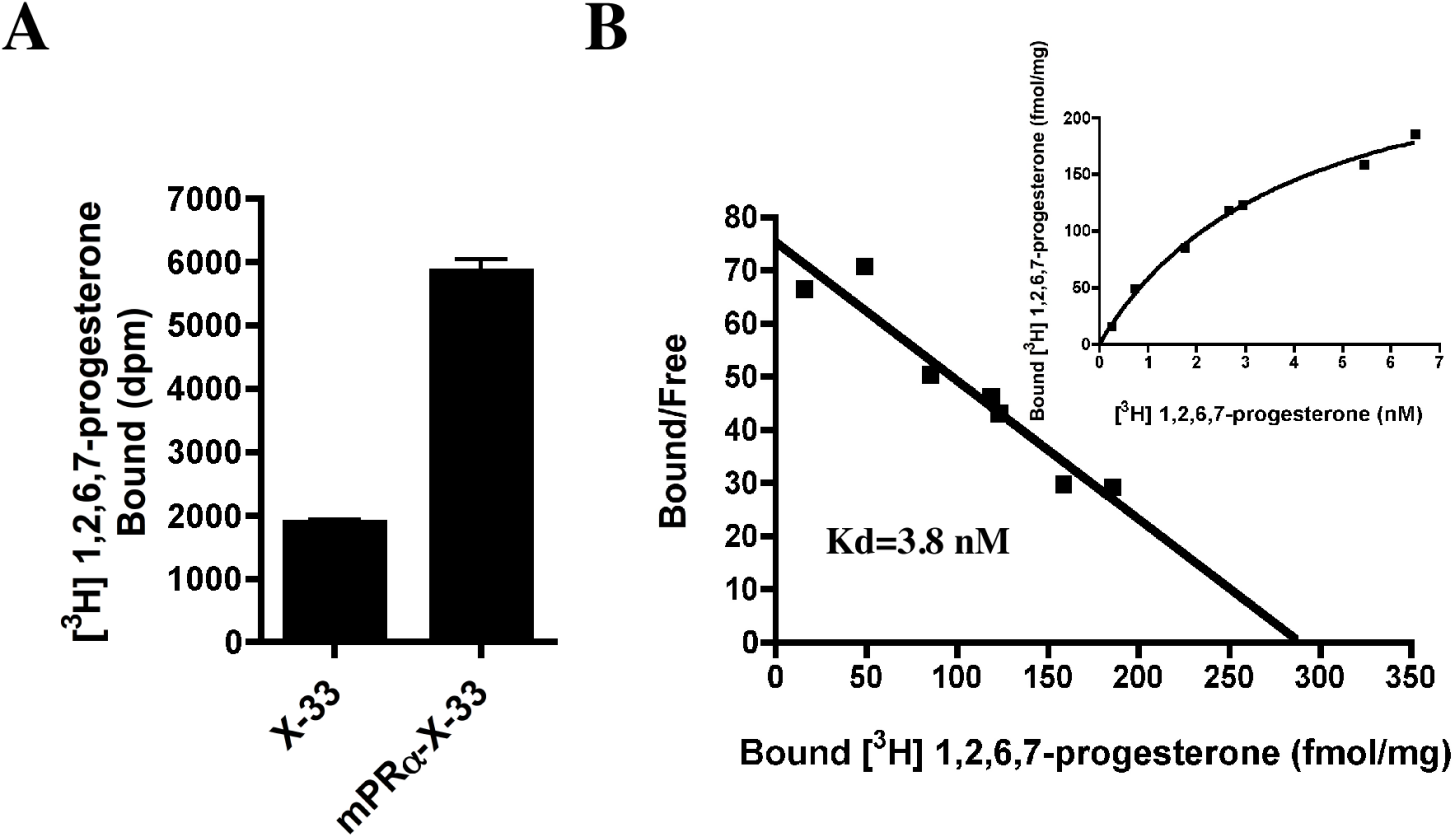
Characterization of binding activity in membrane fractions. (A) Specific binding activity of [^3^H]1,2,6,7-progesterone to membrane preparations from untransformed yeast cells (X-33) and hmPRα-producing cells (mPRα-X33). (B) Saturation curves and Scatchard plots of specific [^3^H]1,2,6,7-progesterone binding to membrane preparations from hmPRα-producing cells (mPRα-X33).

To demonstrate specific binding of [^3^H]1,2,6,7-progesterone to the expressed hmPRα protein, digitonin was used for the preparation of the cell membrane fraction because this glycoside facilitates steroid receptor access [25,26]. Previously, a final concentration of 0.1% digitonin was reported to be optimal for facilitating steroid binding [24], which was measured using a filter-binding assay [25,26]. After the treatment of the crude cell membrane fractions with 0.1% digitonin, the specific [^3^H]1,2,6,7-progesterone-binding activity was significantly increased in membrane fractions from hmPRα-expressing cells. Under the same conditions, fractions from untransformed host cells exhibited lower binding activity (Fig 2A). Saturation analysis demonstrated that the progesterone binding to the cell membranes of hmPRα-expressing cells is saturable and of limited capacity (Bmax = 288.8 fmol/mg). Scatchard analysis indicated the presence of a single site of high-affinity binding sites (Kd = 3.8 nM) in the cell membrane fraction of hmPRα (Fig 2B). Consequently, these results indicated that the heterologously produced recombinant hmPRα was active.

### 3. Solubilization and purification of hmPRα protein

To lyse large amount of yeast cells, a new technology was applied. We applied Ball Mill equipment (Ball Mill PM 100) that can disrupt the samples by rotating stainless steel balls in the chamber under freezing conditions [27,28]. By using this machine, we could homogenize cell precipitates from a 500 ml culture all at once. After the disruption of the yeast cells by the Ball Mill PM 100 instrument, the membrane proteins were solubilized using 0.1% n-dodecyl-β-D-maltoside (DDM), as described previously [24]. To optimize the conditions for Ni-NTA affinity chromatography, 16 lysis buffers (50 mM NaH2PO4, 300 mM NaCl, 1 mM PMSF, 10% glycerol, 0.1% DDM) with 4 different concentrations of imidazole (10, 20, 40 or 80 mM) and of pH 5.0, 6.0, 7.0 or 8.0 were tested (S1 Fig). Membrane preparations were incubated with one of the 16 different lysis buffers for 30 min on ice, and then the solubilized supernatant was separated from insoluble materials by centrifugation (20,000 × *g*, 4°C, 20 min). The solubilized hmPRα fraction was applied to the Ni-NTA resin. Unbound materials in the Ni-NTA resin were subsequently separated by centrifugation, and bound proteins were eluted by elution buffer. The remaining materials were solubilized by denaturing buffer for SDS-PAGE. The hmPRα content in each fraction was analyzed by western blotting using anti-His-tag antibodies. Out of the 16 buffers tested, the 40 mM imidazole, pH 6.0, lysis buffer demonstrated the best separation of hmPRα from other proteins (S1 Fig). Thus, this buffer was selected as the lysis buffer and as the Ni-NTA chromatography running buffer.

**Fig 3:**
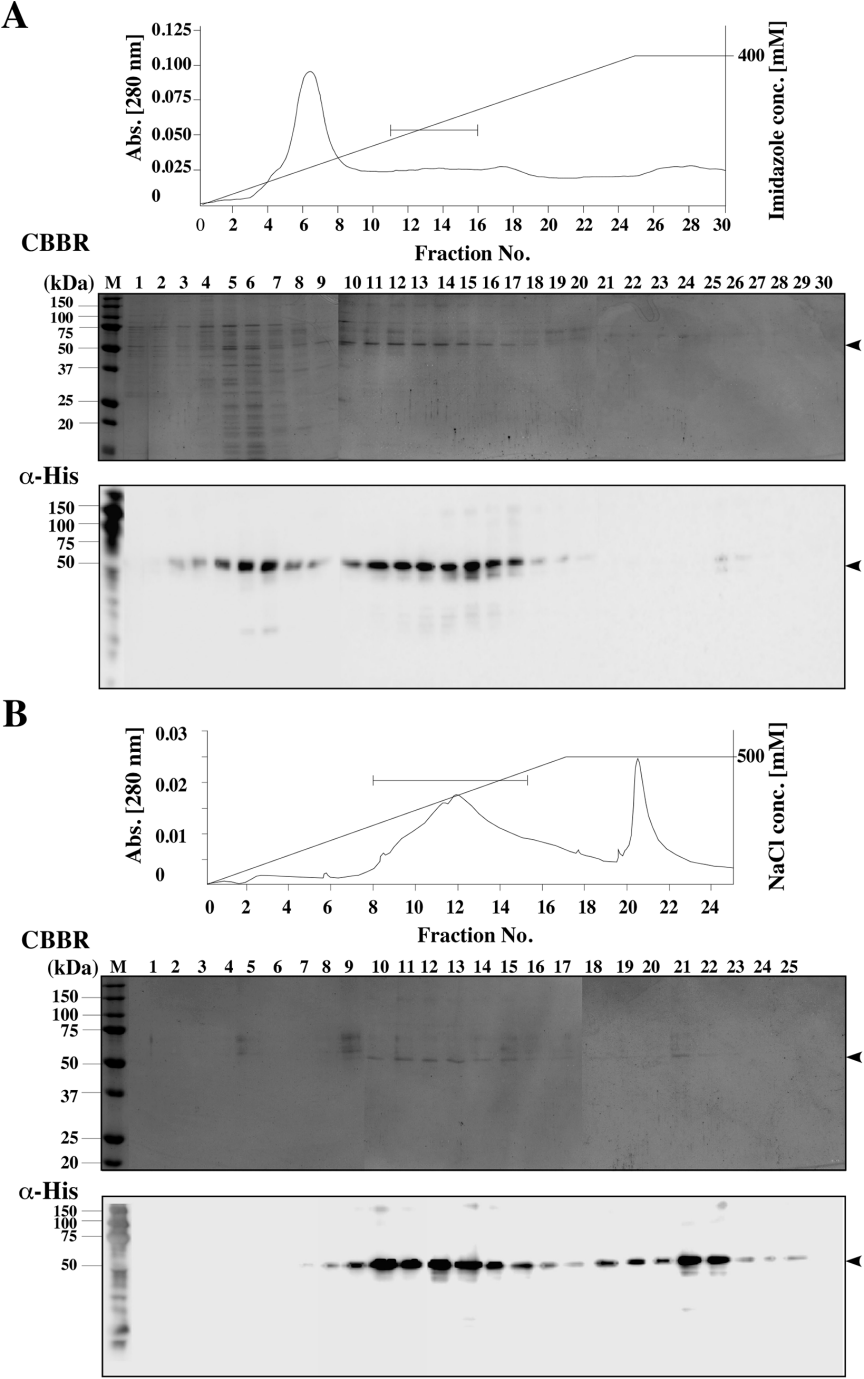
Purification of hmPRα protein by Ni-NTA and amino cellulose column chromatography. (A) Chromatogram and the SDS-PAGE and the western blot analysis results of the Ni-NTA column chromatography fractions obtained from the first purification step. (B) Chromatogram and the SDS-PAGE and western blot analysis results from the Cellufine Amino column chromatography conducted as the second purification step. The elution profile was monitored by absorbance at 280 nm. The horizontal bars in the chromatogram represent the fractions collected for further steps.

**Fig 4:**
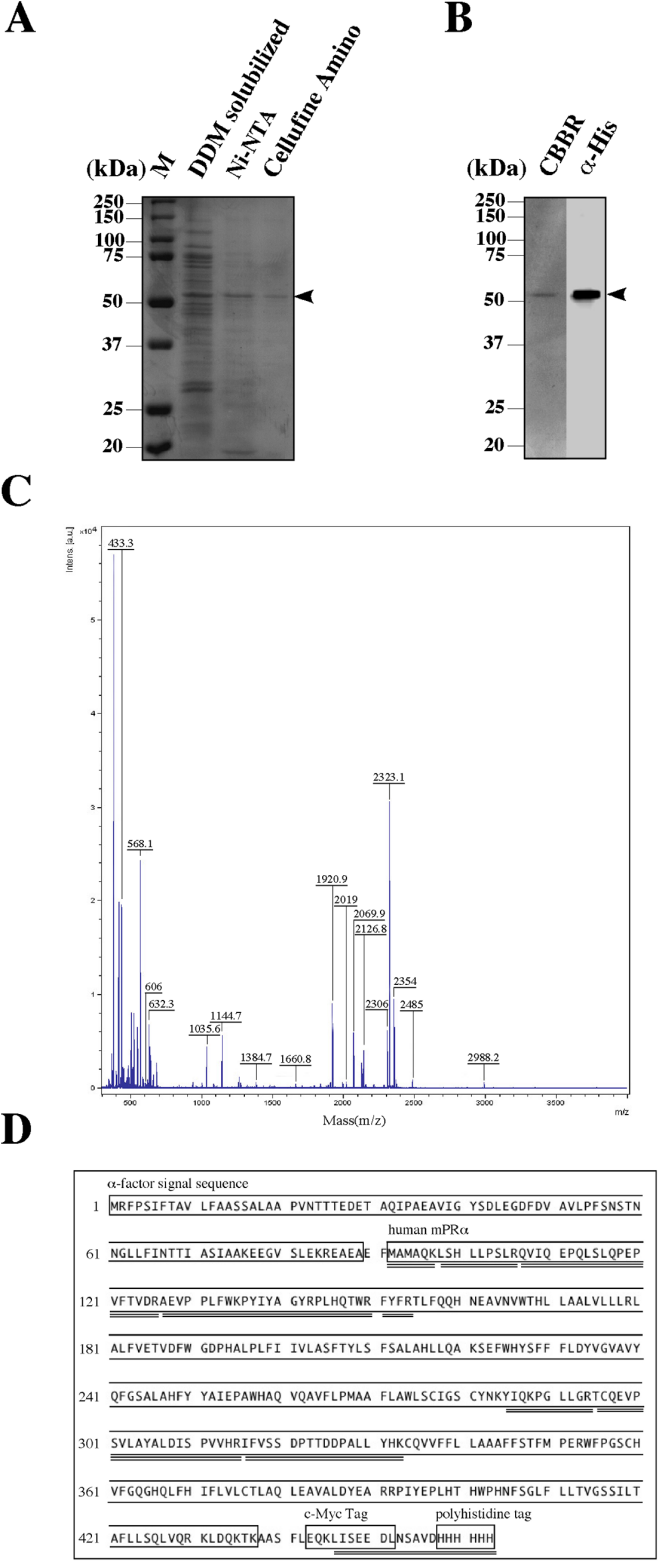
Identification of purified recombinant protein as mPRα. (A) SDS-PAGE analysis of representative fractions after solubilization of the membrane preparation (DDM solubilized), column chromatography over Ni-NTA, amino cellulose (Cellufine Amino). Protein bands were detected by CBBR staining. (B) SDS-PAGE analysis of purified hmPRα. Protein bands were detected by CBBR staining (CBBR) or were immunostained by anti-His-tag antibody (α-His). An arrow indicates hmPRα. (C) MALDI-TOF mass spectrum of purified hmPRα. (D) Amino acid sequence of recombinant hmPRα from produced in this study. The matching peptides from the peptide mass fingerprint analysis are underlined. The sequences of α-factor signal, c-Myc-tag and His-tag are boxed.

In the first step of purification, the sample was separated on a Ni-NTA column. The protein content of the eluted fractions were analyzed by CBBR and immunoblotting with anti-His-tag antibodies. HmPRα protein was detected in fractions 11 to 16 (Fig 3A), which corresponded to 160 mM imidazole in the buffer. These fractions were pooled and applied to a Cellufine Amino column, which we selected as an effective resin for purification of the mPRα protein [24]. The proteins were eluted by linear gradient of sodium chloride (Fig 3B). In the third purification step, the hmPRα fractions were passed through a SP-Sepharose column. The purified hmPRα proteins were concentrated using Cellufine Amino resin. The SDS-PAGE and immunoblotting assay indicated that hmPRα was successfully purified with higher purity (Fig 4A).

### 4. Characterization of purified recombinant hmPRα

**Fig 5:**
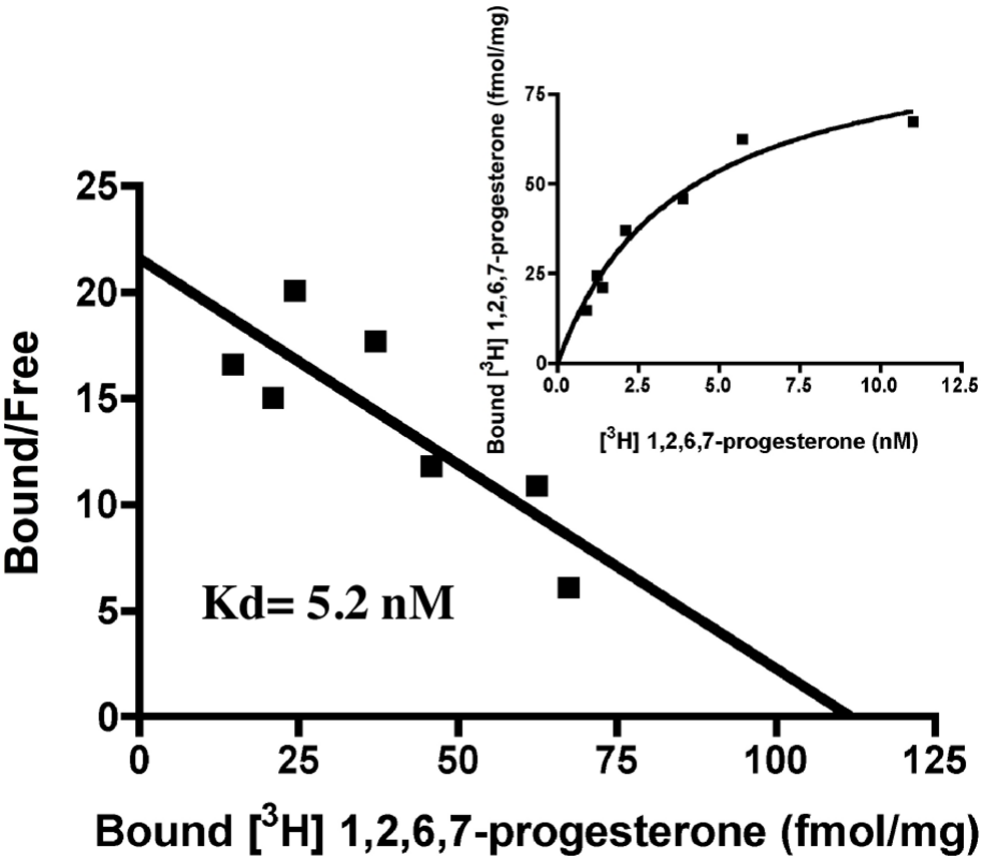
Scatchard plot analysis of purified hmPRα. Saturation curves and Scatchard plots of specific [^3^H]1,2,6,7-progesterone binding to purified recombinant hmPRα.

The identity of the purified proteins was confirmed by MALDI-TOF/MS analysis. Peptide mass fingerprint analysis of the purified 50-kDa protein confirmed the presence of hmPRα (Fig 4B and 4C). From the results, the hmPRα protein was concluded to have been successfully expressed and purified. To examine the binding activity of the purified hmPRα, we modified the steroid binding assay for solubilized mPR proteins. When Ni-NTA was added into the reaction mixture, the steroid-binding activity of hmPRα could be detected (S2 Fig). Using this method, specific progesterone-binding activity was detected in the purified hmPRα fraction. Scatchard analysis indicated the presence of a single class of high-affinity binding site (Kd = 5.2 nM) with limited capacity (Bmax = 111.6 fmol/mg) (Fig 5).

## Discussion

Previously we reported the expression and purification of goldfish mPRα in a *P*. *pastoris* yeast host system [24]. Based on the procedure established for goldfish mPRα, we succeeded in producing and purifying a relatively large amount of recombinant human mPRα protein in this study. To obtain a large amount of product, we optimized the yeast culture and buffer conditions for Ni-NTA column chromatography. Furthermore, a new method of disrupting yeast cells using a ball mill was applied [27,28]. Using the ball mill, a membrane fraction with higher mPRα protein content could be obtained. The amount of expressed hmPRα was determined to be approximately 150 mg/L of culture by Western blot analysis. Of this amount of expressed protein, we could purify 1.2–1.5 mg of hmPRα with a yield of 0.8–1.0%. The yield of activity was increased by more than 100 times compared to that of goldfish mPRα expression and purification (Table 1).

**Table 1:** Summary of the purification of recombinant human mPRα from *Pichia pastoris*.

	Protein (mg)	Total activity^a^ Bound [^3^H] 1,2,6,7 progesterone (nmol)	Specific activity (pmol/mg)	-fold	Yield^b^ (%)
Crude extract	1264.5	0.534	0.42	1.0	100.0
DDM solubilization	197.2	0.232	1.18	2.8	43.4
Ni-NTA	68.4	0.104	1.52	3.6	19.5
Cellufine Amino	4.5	0.015	3.33	7.9	2.8
SP-Sepharose	1.2	0.012	10.00	23.8	2.2

^a^ The activities of each fraction were measured with 4 nM of [^3^H] 1,2,6,7 progesterone as described in Materials and Methods.

^b^ The yield of total progesterone binding activities of each fraction are presented as percentages of the crude extract, which was assumed to have a binding activity of as 100%.

The progesterone-binding assay demonstrated that the hmPRα was expressed in an active form. The steroid-binding activity of membranes prepared from mPRα-expressing *P*. *pastoris* cells was detected in the presence of digitonin. The effect of digitonin was described in bovine membrane fractions [25]. In presence of digitonin, the binding of the membrane fraction of human sperm to progestin has also been detected [26]. A similar effect was confirmed in our previous study of goldfish mPRα [24]. The progesterone-binding assay using purified hmPRα demonstrated that the protein remained active after solubilization and purification (Fig 5). To measure the steroid-binding activity of solubilized hmPRα, we supplemented Ni-NTA resin into the reaction mixture, which trapped the protein on the filter (S2 Fig). Via this modification, we succeeded in measuring the steroid binding activity of purified hmPRα. The Kd value of purified hmPRα did not change significantly, compared with the value for the membrane fraction. Thus, we can conclude that we established a procedure to purify hmPRα protein in an active form.

mPRs have been identified in a wide variety of organisms ranging from fish to humans [29,30], and progestin-binding activity in these species have been reported in many species, including fish (goldfish, seatrout and zebrafish), frogs and mammals (cattle, rats, mice and humans) [8,31–33]. mPRα was found to be expressed in the reproductive tissues (ovary, uterus and testes), kidneys, brain and spinal cord in vertebrates [34–36]. The broad distribution of mPRs suggests that these proteins play a role in a wide variety of steroid-related functions in tissues.

Some studies regarding mPRs in the brain have been reported. Whole brain expression analysis of progestin receptors revealed that mPRα and β were expressed in thalamic nuclei [37]. The results suggested that mPRs are involved in the regulation of sensory and cognitive functions. The expression of 5 subtypes of mPRs, including two new subtypes of mPRs, δ and ε, was analyzed by q-PCR in human brain [13]. Among the mPR subtypes, mPRε was the most abundant subtype in the brain and is a potential intermediary of the antiapoptotic effects of neurosteroids in the central nervous system. The roles of brain mPRs in the regulation of mammalian behavior have also been investigated [38,39].

Progesterone signaling through mPRs in human breast cancer cells has been investigated [40]. Zuo, L. et al. suggested that progesterone promotes epithelial-to-mesenchymal transition of breast cancer cells through mPRs [41]. The gene expression level of mPRα has been as a biomarker for breast cancer survival [42]. Recently, progesterone has been demonstrated to generate cancer stem cells through mPRs in mammary cells [43].

Progesterone may interact with mPRα, mPRβ and mPRγ, which implies negative consequences on the proliferation of human T-cells that may attack fetuses during pregnancy, as indicated by changes in pH and Ca^2+^ levels inside T-cells [44]. Moreover, progesterone may be involved with bovine T-lymphocyte activation and proliferation through binding to mPRs in the corpus luteum [45]. Progesterone signaling in murine macrophages is associated with parturition that may be regulated by mPRα. This relationship may contribute to the functional withdrawal of progesterone associated with labor [46].

These studies have drawn attention to the discovery of novel drugs or treatment of diseases such as reproductive problems, cancers and encephalitis.

In this study, we established an hmPRα-expressing yeast strain and a method to purify a large amount of active mPRα protein. The purified active hmPRα could be applicable for the screening of ligands for hmPRα. Additionally, endocrine-disrupting chemicals that interact with hmPRα could be identified by this screening. Very recently, the three-dimensional structure of PAQR1 was reported [47]. hmPRα belongs to a GPCR family, progestin and adipoQ receptor family (PAQR family), which is composed of 11 genes. The three-dimensional structure of crystallized adipoQ receptor 1 (PAQR1) has been resolved. A novel class of proteins with a seven-transmembrane domain structure and a zinc-binding cavity was discovered. The strategy of structure resolution could be applied to mPRs. The recombinant protein expressed in this study will be useful for such an approach.

Recently, a new member of the membrane steroid receptor family was identified [48,49]. Through screening using monoclonal antibody for membrane proteins, a cDNA for a membrane androgen receptor (mAR) was identified as the previously reported zinc transporter ZIP9 subfamily (SLC39A9). Similar to mPRs, a seven-transmembrane domain structure was predicted from the amino acid sequence of ZIP9. ZIP9 is widely expressed in human tissues and upregulated in malignant breast and prostate tissues, suggesting that it is a potential therapeutic target for treating breast and prostate cancers. mAR has been found to be a seven-transmembrane domain receptor for three types of steroids (progestin, estrogen and androgen). All of these receptors are conserved among vertebrates and are widely distributed in various tissues. Thus, these findings suggest that tissues are regulated by the nongenomic activity of various types of steroids. The distinct roles of steroidal nongenomic effects in various tissues should be addressed.

## Methods

### Ethics Statement

The present study using human gene was approved by the Research Ethics Review Committee regarding Human Subjects of the Shizuoka University.

### Materials

[^3^H]1,2,6,7-progesterone was purchased from PerkinElmer Inc. The modified trypsin (sequencing grade) was from Promega (Tokyo, Japan). The CHCA was obtained from Bruker Daltonics (Billerica, MA). Digitonin was purchased from Sigma-Aldrich Chemicals (St. Louis, MO). The DNA polymerase and DNA Ligation Kit were from Takara Bio (Siga, Japan). The DNA fragment extraction kit from agarose gel was purchased from QIAGEN (Tokyo, Japan). The molecular weight marker for SDS-PAGE was from Bio-Rad (Hercules, CA). The anti-rabbit antibody conjugated with peroxidase and yeast nitrogen base without amino acids were obtained from Invitrogen (Carlsbad, CA). The anti-His-tag antibody was from Medical & Biological Laboratories (Nagoya, Japan). Other chemicals were purchased from Wako Pure Chemical Industries, Ltd. (Osaka, Japan).

### Construction of recombinant pPICZαA plasmid

Human mPRα protein was expressed in *P*. *pastoris* using the wild strain X33. The cDNA for hmPRα was prepared from human blood and amplified by polymerase (KOD plus neo, TOYOBO, Japan) using a primer set of Hs mPRα normal F, GTCACCTGGCTTTGCCTTTG, and Hs mPRα normal R, ATGCCATCCCCCTTCACTTG. Then, the amplified DNAs were inserted into a pBluescript II KS(+) plasmid and transformed into *E*.*coli* (XL1 Blue) for the cloning of the hmPRα gene. After the completion of cloning, the hmPRα fragment of the pBluescript II KS(+) plasmid was also amplified as a DNA template by polymerase (KOD plus neo, TOYOBO, Japan) using the primer set of Hs mPRα EcoRI, CGGAATTCATGGCCATGGCCCAGAAACTCAGCCACCTCCTGCCGAG, and Hs mPRα-NotI, ATAAGAATGCGGCCGCCTTGGTCTTCTGATCAAGTTTGCGCTGTACCAGC. For the expression in *P*. *pastoris*, the DNA was inserted into the *P*. *pastoris* expression vector pPICZαA (Invitrogen). All of the ORF region DNA sequences of the expression vectors were verified by DNA sequencing.

The *P*. *pastoris* strain X-33 (Invitrogen) was transformed with the hmPRα-expression construct by electroporation, as previously described in detail [24]. The construct was linearized with PmeI digestion, and the linearized plasmid (168 μg) was used to transform *P*. *pastoris* cells through electroporation. Electroporation was performed using a Gene Pulser instrument (Bio-Rad), following previously established protocols [24].

Yeast extract-peptone-dextrose medium (YPD) plates (1% yeast extract, 2% peptone, 2% dextrose, 2% agar) containing 500 μg/mL Zeocin were selected for the culture of the recombinant colonies. The genomic amalgamation of hmPRα construct was verified by PCR using Ex Tag Polymerase (Takara Bio, Siga, Japan) and the primer set of 5′AOX1, GACTGGTTCCAATTGACAAGC, and 3′AOX1, GCAAATGGCATTCTGACATCC, to amplify the sequence between the AOX1 promoter and terminator regions (Fig 1A). The production of recombinant protein was confirmed by analyzing several Zeocin-resistant clones. The clones with the highest expression levels were maintained and stored on MD plates contained 1.34% yeast nitrogen base, 4 x 10^−5^% biotin, 2% dextrose, 1.5% agar [24] at 4°C.

### Expression of hmPRα in *P*. *pastoris*

A single colony expressing hmPRα from the MD plate was inoculated in 100 ml of BMGY medium (1% yeast extract, 2% bactopeptone, 100 mM potassium phosphate, pH 6.0, 1.34% yeast nitrogen base without amino acids, 4 x 10^−5^% biotin, 1% glycerol) and incubated for 21 hours at 30°C with shaking at 180 rpm. The volume was increased to 500 mL of BMGY medium in a 2 L baffled flask, and the yeast was incubated for 16.5 hours at 30°C with shaking at 180 rpm until the OD_600_ nm reached 17–19. A 1 ml aliquot of the culture medium was used to determine the cell density. The remaining culture was harvested by centrifugation at 3,000 x *g* for 5 min and was washed once using 300 ml BMMY medium. For the induction of mPRα protein expression, the cells were resuspended in 400 ml BMMY (1% yeast extract, 2% bactopeptone, 100 mM potassium phosphate, pH 6.0, 1.34% yeast nitrogen base without amino acids, 4 x 10^−5^% biotin, 0.5% methanol) to an OD_600_ of 21–23. The medium was placed in a 2 L baffled flask and incubated at 20°C for 6 hours with shaking at 180 rpm. After 6 hours, the cells were harvested by centrifugation at 3,000 × *g* for 5 min, and the precipitate was frozen with liquid nitrogen and stored at -80°C.

### Membrane preparation and solubilization of membrane proteins

Frozen cell pellets (≅20 g) that were harvested from 800 ml of culture were thawed and resuspended in 80 ml of ice-cold lysis buffer (50 mM sodium phosphate, 1 mM PMSF, 1 mM EDTA, 5% glycerol, pH 7.4). Then, re-frozen as shape of tubules in the stainless chamber for cell breaking with stainless ball. Consecutively, cells were broken by Retsch Ball Mill PM 100 (Verder Scientific Co., Ltd., Haan, Germany) with six rounds of shaking at fixed 400 rpm for 3 min with an interval of chilling with liquid nitrogen. Then, the disrupted cells were collected into centrifuge tubes. Nonhomogenized cells and debris were separated from the fractions containing the membranes by low-speed centrifugation (1,000 x g, 4°C, 7 min). After the supernatant collection, the pellet was resuspended in 30 ml of ice-cold lysis buffer for a further round of supernatant collection. The supernatants were combined, and the membrane fractions were recovered by centrifugation at 20,000 × *g*, 4°C, for 20 min. The precipitates were resuspended in buffers for the steroid binding assay or purification based on their intended use.

### Purification

To purify the hmPR, the solubilized proteins were thawed on ice and loaded onto a 80 mL Ni-NTA Agarose (QIAGEN, Gaithersburg, MD, USA) column (φ 4.5 × 5.0 cm) that was equilibrated with lysis buffer containing 0.01% DDM and 1 mM PMSF. The proteins were eluted with a 500 mL gradient of 10–400 mM imidazole in the same buffer and washed with 100 ml of the same buffer containing 400 mM imidazole. The fractions that contained recombinant hmPRα were identified by western blot analysis and were collected and diluted for 4.5 times with DDW. Then samples were loaded onto a 5 mL of Cellufine Amino (JNC Corporation, Tokyo, Japan) column (φ1.6 × 10 cm) that was equilibrated with CA buffer (50 mM Tris-HCl buffer, pH 8.0, containing 0.01% DDM and 1 mM PMSF). The column was washed with 15 ml of the same buffer and eluted with a 120 mL gradient of 0–0.5 M NaCl in CA buffer. Fractions containing hmPRα were collected and were then passed through a SP-Sepharose column (1 ml) and applied to 1.5 ml of a Cellufine Amino column. The proteins were eluted with CA-buffer containing 0.5 M NaCl. The fractions that contained the hmPRα protein were collected and concentrated with Centriprep YM-3 filter units (Millipore, Billerica, MA).

### SDS-PAGE and western blot analysis

Proteins were separated by SDS-polyacrylamide gel electrophoresis (SDS-PAGE) on a 12% polyacrylamide gel under denaturing conditions according to the method of Laemmli and were transferred to Immobilon membranes (Millipore, Billerica, MA). The membranes were blocked in 5% nonfat powdered milk in 20 mM Tris-buffered saline, pH 7.6 (TBS) containing 0.1% Tween 20 (TTBS) for 1–2 hours at room temperature. Then, the membranes were incubated with primary antibodies (1,000-fold dilution in TBS buffer) and with secondary antibodies (2,000-fold dilution in TBS buffer). The visualization of the target protein was performed by enhanced chemiluminescence using an ECL detection kit (PerkinElmer, Waltham, MA), a method based upon a chemiluminescent reaction mediated by peroxidase conjugated to a secondary antibody. The signals were digitized using a CCD camera system (Luminescent Image Analyzer LAS-4000 mini; Fujifilm, Tokyo, Japan).

### Peptide mass fingerprint analysis by MALDI-TOF/MS

Purified recombinant mPRα proteins stained with CBBR in SDS-PAGE gel slices were trypsinized. Subsequently, the peptides were recovered using a ZipTip (Millipore) and were eluted through a solution (2 μl) containing 60% acetonitrile, 0.1% TFA and 5 mg/ml of CHCA (Bruker Daltonics), as described previously for goldfish mPRα [24]. A 384-well plate was used for the loading of the samples, which was contained a double layer with CHCA and dissolved in acetone, after that air-dried. A MALDI-TOF/MS Autoflex (Bruker Daltonics, Billerica, USA) was used to detect the peptide mass spectrum in a positive ion mode. The spectra that were obtained from MALDI-TOF/MS were calibrated by a mixture of molecular weight standards (Bruker Daltonics). By using the MASCOT software (Matrix Science, London, UK), the peptide fingerprint was analyzed and was compared with peptides from human taxonomy using the NCBInr database. Subsequent parameters used included cysteine modification by carbamidomethylation (C), a trypsin digest missed cleavage of zero and a peptide mass tolerance ± 0.4 Da. The mPRα protein was identified from the molecular weight of peptide fragments using probability-based MOWSE scores.

### Radiolabeled ligand binding assays

The plasma membrane pellet was obtained as described in the membrane preparation and solubilization section. Then, the pellet was resuspended in HEAD buffer (25 mM HEPES, 10 mM NaCl, 1 mM dithiothreitol, 1 mM EDTA, pH 7.6) containing 0.1% digitonin. Progestin receptor binding to the membrane fractions was measured following previously established procedures [20]. In the binding assay for the solubilized samples, Ni-NTA resin (100 μl of 50% vol) was added. GF/B filters were presoaked in wash buffer without Tween 80.

### Competition studies

One set of tubes contained 1.5 nM [^3^H]1,2,6,7-progesterone alone (total binding); another set also contained cold progestin competitor at a 100-fold greater concentration to measure nonspecific binding (NSB). After a 30 min incubation at 4°C with the membrane fractions, the reaction was stopped by filtration (Whatman GF/B filters, presoaked in wash buffer containing 2.5% Tween 80). The filters were washed three times with 5 mL of wash buffer (25 mM HEPES, 10 mM NaCl, 1 mM EDTA, pH 7.4) at 4°C, and the bound radioactivity were measured by scintillation counting.

### Saturation analyses and Scatchard plots

Various concentrations (0.5–12.5 nM) of [^3^H]1,2,6,7-progesterone (specific activity, 96.6 Ci/mmol) were added to the assay tubes with (nonspecific) or without (total) 100-fold molar excess cold progesterone. Linear and nonlinear regression analyses for all receptor binding assays and calculations of K_d_ and binding capacity (Bmax) were conducted using GraphPad Prism for Macintosh (version 4.0c; GraphPad Software, San Diego, CA). The results are shown as Scatchard plots.

## Supporting Information

S1 Fig Optimization of conditions for Ni-NTA column chromatography. Binding of solubilized mPRα onto the Ni-NTA resin was examined with different concentrations of imidazole (10, 20, 40 or 80 mM) and pH values (pH 5.0, 6.0, 7.0, or 8.0) in Ni-NTA binding buffer (50 mM NaH2PO4, 300 mM NaCl). Samples for each lane are following; M, marker; S, solubilized mPRα protein fraction; T, flow-through protein after Ni-NTA binding; E, eluted proteins with elution buffer (50 mM NaH2PO4, 300 mM NaCl, 250 mM imidazole pH 8.0); R, remained on Ni-NTA resin after elution. The proteins were detected by CBBR staining (upper panel in each set) or western blotting (lower panel in each set). The panels depict the results obtained using (A) 10 (B) 20 (C) 40 and (D) 80 mM imidazole-containing buffer of various pH levels.
(TIF)

S2 Fig Optimization of the attachment of purified mPRα with Whatman UK GF/B filters for [^3^H]1,2,6,7-progesterone-binding assay analysis. (A) The indicated amount of Ni-NTA resin (10, 20, 50 or 100 μl) was supplemented into the reaction mixture of the steroid binding assay. After filtration, the mPRα protein content remaining on the filter or present in the flow-through was determined by Western blot analysis using α-His-tag antibody. (B) Specific binding activity of purified mPRα to [^3^H]1,2,6,7-progesterone with 10 and 100 μl Ni-NTA resin supplemented in the reaction mixture.
(TIF)
