# Supplementary figures and images for: Expression and Purification of Human Membrane Progestin Receptor α (mPRα)
Source: PLoS One. 2015 Sep 23;10(9):e0138739. doi: 10.1371/journal.pone.0138739 (PMC4580469; doi:10.1371/journal.pone.0138739)

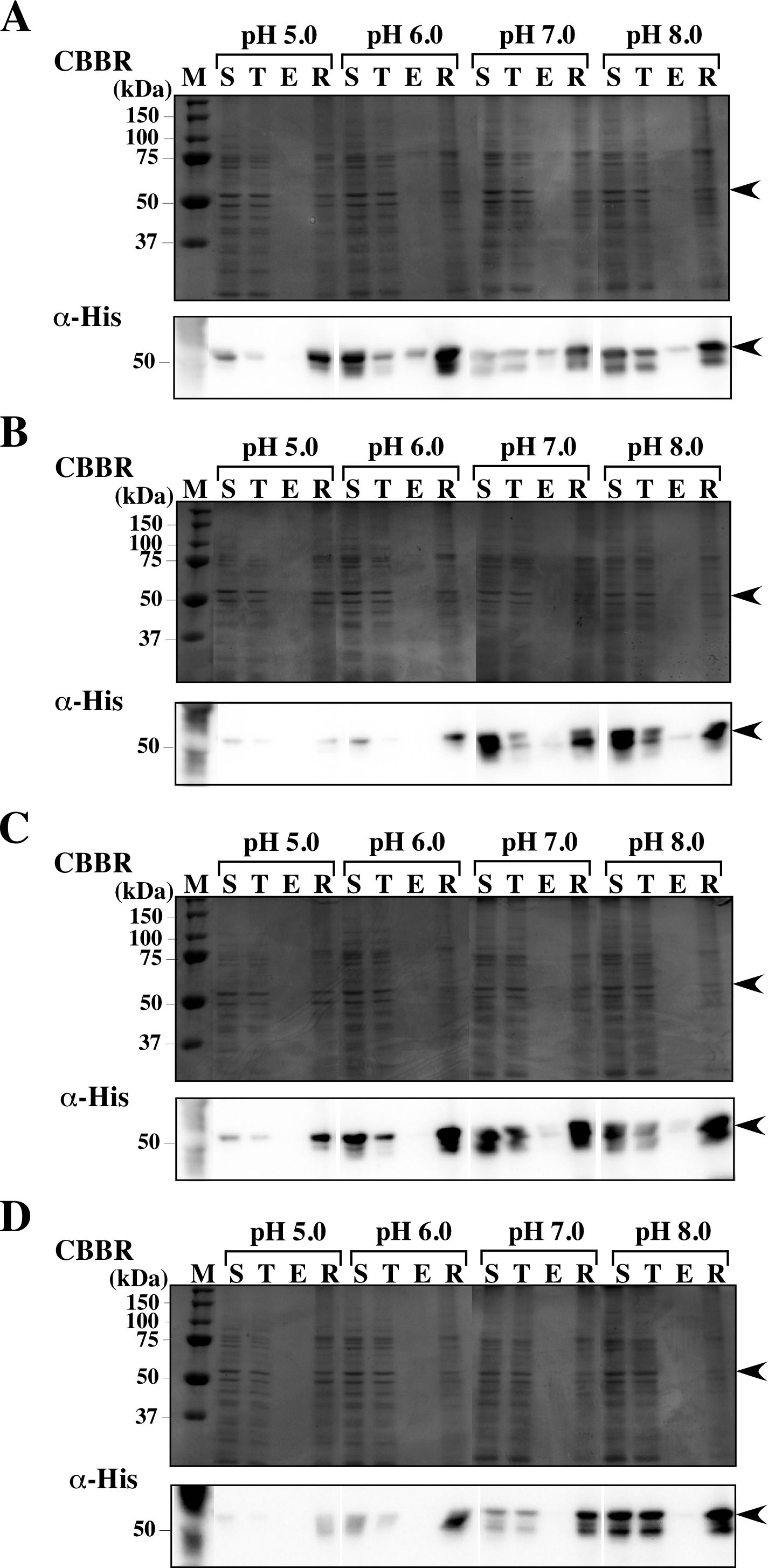

Supplement: S1 Fig — Binding of solubilized mPRα onto the Ni-NTA resin was examined with different concentrations of imidazole (10, 20, 40 or 80 mM) and pH values (pH 5.0, 6.0, 7.0, or 8.0) in Ni-NTA binding buffer (50 mM NaH2PO4, 300 mM NaCl). Samples for each lane are following; M, marker; S, solubilized mPRα protein fraction; T, flow-through protein after Ni-NTA binding; E, eluted proteins with elution buffer (50 mM NaH2PO4, 300 mM NaCl, 250 mM imidazole pH 8.0); R, remained on Ni-NTA resin after elution. The proteins were detected by CBBR staining (upper panel in each set) or western blotting (lower panel in each set). The panels depict the results obtained using (A) 10 (B) 20 (C) 40 and (D) 80 mM imidazole-containing buffer of various pH levels. (TIF) [file pone.0138739.s001.tif]

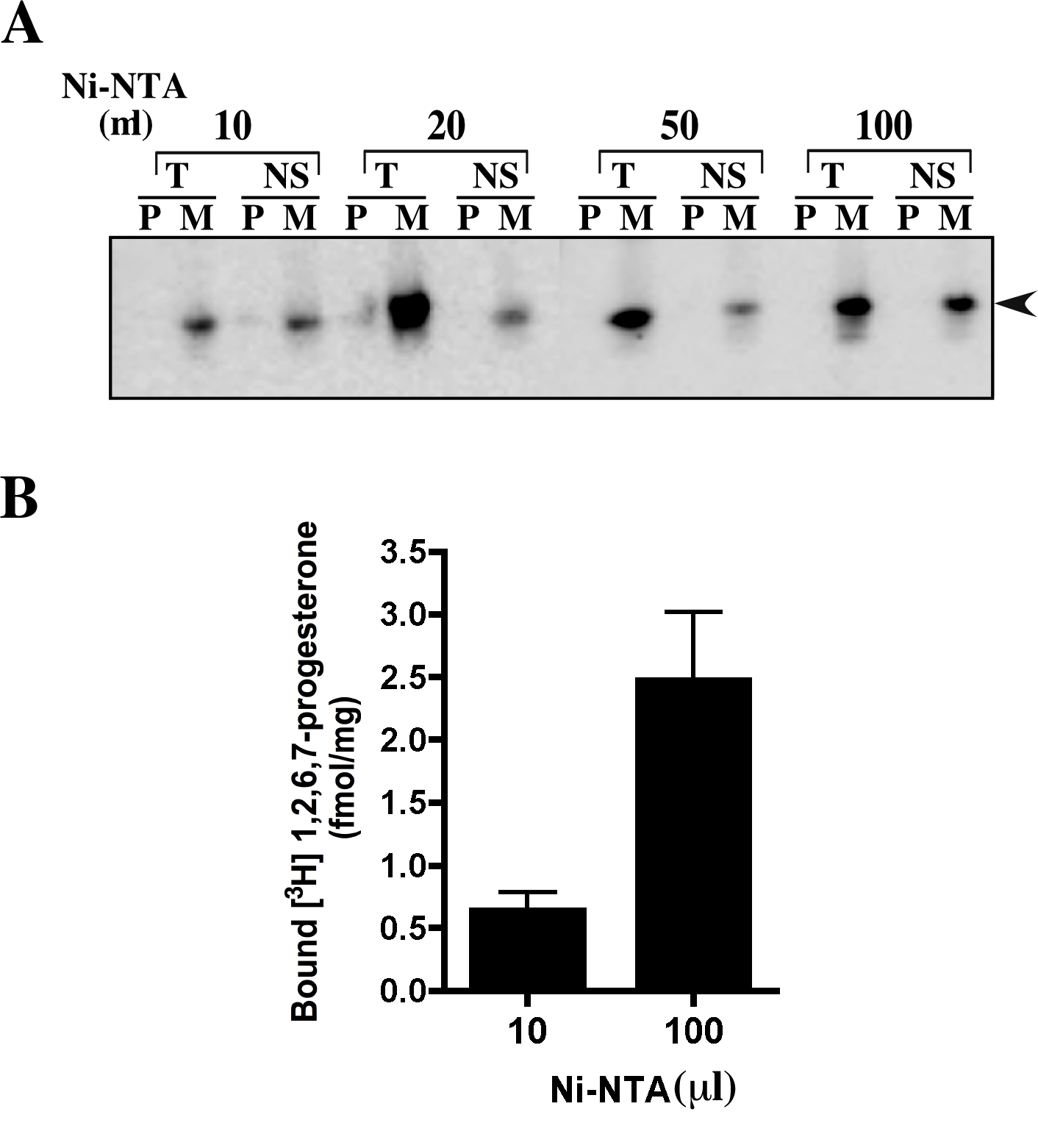

Supplement: S2 Fig — (A) The indicated amount of Ni-NTA resin (10, 20, 50 or 100 μl) was supplemented into the reaction mixture of the steroid binding assay. After filtration, the mPRα protein content remaining on the filter or present in the flow-through was determined by Western blot analysis using α-His-tag antibody. (B) Specific binding activity of purified mPRα to [3H]1,2,6,7-progesterone with 10 and 100 μl Ni-NTA resin supplemented in the reaction mixture. (TIF) [file pone.0138739.s002.tif]
